# Simultaneous Formate and Syngas Conversion Boosts Growth and Product Formation by *Clostridium ragsdalei*

**DOI:** 10.3390/molecules29112661

**Published:** 2024-06-04

**Authors:** Irina Schwarz, Angelina Angelina, Philip Hambrock, Dirk Weuster-Botz

**Affiliations:** Chair of Biochemical Engineering, School of Engineering and Design, Technical University of Munich, Boltzmannstr. 15, 85748 Garching, Germany; irina.schwarz@tum.de (I.S.);

**Keywords:** *Clostridium ragsdalei*, circular carbon economy, C1 carbon sources, acetogens, syngas fermentation, biofuels

## Abstract

Electrocatalytic CO_2_ reduction to CO and formate can be coupled to gas fermentation with anaerobic microorganisms. In combination with a competing hydrogen evolution reaction in the cathode in aqueous medium, the in situ, electrocatalytic produced syngas components can be converted by an acetogenic bacterium, such as *Clostridium ragsdalei*, into acetate, ethanol, and 2,3-butanediol. In order to study the simultaneous conversion of CO, CO_2_, and formate together with H_2_ with *C. ragsdalei*, fed-batch processes were conducted with continuous gassing using a fully controlled stirred tank bioreactor. Formate was added continuously, and various initial CO partial pressures (pCO_0_) were applied. *C. ragsdalei* utilized CO as the favored substrate for growth and product formation, but below a partial pressure of 30 mbar CO in the bioreactor, a simultaneous CO_2_/H_2_ conversion was observed. Formate supplementation enabled 20–50% higher growth rates independent of the partial pressure of CO and improved the acetate and 2,3-butanediol production. Finally, the reaction conditions were identified, allowing the parallel CO, CO_2_, formate, and H_2_ consumption with *C. ragsdalei* at a limiting CO partial pressure below 30 mbar, pH 5.5, n = 1200 min^−1^, and T = 32 °C. Thus, improved carbon and electron conversion is possible to establish efficient and sustainable processes with acetogenic bacteria, as shown in the example of *C. ragsdalei.*

## 1. Introduction

The steady increase in greenhouse gas emissions in the atmosphere is a leading global issue that has received more attention and awareness in recent years [1,2,3]. In regards to climate change, establishing a circular carbon economy with CO_2_ emission reduction is an objective in the chemical industry since fossil raw material still serves for the production of carbonaceous chemicals [4,5]. Syngas fermentation using acetogenic bacteria can be a biotechnological alternative to substitute fossil carbon-based chemical synthesis processes [6,7]. In this case, acetogenic microorganisms can use CO, CO_2_, and H_2_ as carbon and electron sources for growth and product formation, such as organic acids, their corresponding alcohols, or other value-added organic chemicals. For example, acetate, ethanol, and 2,3-butanediol can be produced autotrophically by the genetically closely related acetogens *Clostridium ljungdahlii* [8,9], *Clostridium autoethanogenum* [10,11], and *Clostridium ragsdalei* [11,12]. Ethanol production by *C. autoethanogenum* using syngas from steel mills has already been established at an industrial scale (https://lanzatech.com/, accessed on 18 April 2024). Other acetogens, for example, *Clostridium carboxidivorans* [13] or *Butyribacterium methylotrophicum* [14], are known to produce butyrate and butanol from syngas.

Medium-chain organic acids, like butyrate, hexanoate, and octanoate, can be produced by chain elongation with anaerobic chain-elongating bacteria, like *Clostridium kluyveri*, through the reverse β-oxidation [15,16] from the main products of the microbial syngas conversion, acetate and ethanol. The co-cultivation of the syngas-converting acetogen *C. carboxidivorans* with chain-elongating *C. kluyveri* enabled the autotrophic production of the medium-chain alcohols 1-butanol, 1-hexanol, and 1-octanol [17]. Strain optimization and metabolic engineering to increase product yields of various short- and medium-chain organic chemicals are ongoing work [18,19,20,21,22]. An example is the carbon-negative process for acetone and isopropanol production by genetically engineered *C. autoethanogenum* [23], which reveals the great potential of syngas fermentation.

Synthesis gas that usually contains CO, CO_2_, H_2_, N_2,_ and trace impurities, depending on the source, can originate from various production processes: steam reforming from natural gas, gasification of biomass, or industrial emissions (e.g., off-gas from steel production). Using the synthesis gas as feed stock for bacteria in gas fermentation can help to reduce CO_2_ emissions and establish a carbon cycle by converting parts of the waste stream into value-added products [4,5,24]_,_ especially when waste gases are only combusted for heat production. Furthermore, mild process conditions, such as low temperatures and ambient pressures, are another advantage over the often energy-consuming chemical synthesis processes [25].

An alternative to produce synthesis gas is CO_2_ electrolysis in the presence of H_2_O for the production of CO and where H_2_ is evolved from protons as a competing electrochemical reaction. Haas et al. [26] demonstrated a scalable approach involving the electrochemical reduction of CO_2_ to CO and protons to H_2_ in the gas phase and transferring the electrolysis syngas into an acetogenic fermentation process. First, *C. autoethanogenum* used the synthesis gas mixture to produce ethanol and acetate. Later, the same bioreactor was inoculated with *C. kluyveri* that synthesized butyrate and hexanoate, and these acids were converted to the corresponding alcohols 1-butanol and 1-hexanol by the remaining *C. autoethanogenum* in the co-culture.

Electrolysis and fermentation can also be integrated into a one-pot process. This was demonstrated by our group using a novel bio-electrocatalytical system (BES), where a zero-gap polymer–electrolyte membrane (PEM) was connected to the bottom of a stirred-tank bioreactor for cathodic CO_2_ reduction [27]. Here, *C. ragsdalei* produced biomass and acetate from electrocatalytically synthesized CO and H_2_.

Besides CO, other gaseous or soluble products can evolve from electrocatalytical CO_2_ reduction depending on the catalyst, reaction conditions, and the catalyst surface structure [28,29]. Examples of soluble by-products are formate and methanol, while methane can evolve as well. In the past, many electrocatalysts were studied for CO_2_ reduction. A silver-based gas diffusion electrode was selective for CO in a gaseous environment, but formate was synthesized as a by-product [26]. Regarding carbon-based catalysts such as M-N-Cs, the coordination of different transition metals within the N-doted carbon structure can form electroactive sites [29,30]. Catalysts with copper can be selective for methane, ethylene, various aldehydes, and formate [31]. Ni, Pd, and Rh as metallic cathodes are selective for formate formation, but CO is synthesized as well [32]. Specifically, formate and methanol as by-products from electrocatalytical CO_2_ reduction are stable and have a high energy content that makes the molecules attractive as (co-)substrates in bacterial fermentation.

To date, formate assimilation has been shown for just a few microorganisms, explaining that pathways for formate fixation are rare [33]. Formate can be oxidized to CO_2_ by the formate dehydrogenase that enters the Calvin cycle for carbon fixation, as shown with *Cupriavidus necator* in the presence of oxygen [34,35] and *Rhodoblastus acidophilus* under anoxic conditions [35,36,37]. Moreover, formate can be reduced in several steps to methylene-tetrahydrofolate (methylene-THF). Methylene-THF reacts with glycine to enter the serine cycle [36,37], and later another CO_2_ is fixed so that finally the main intermediate acetyl-CoA can be formed. The reductive glycine pathway, found in anaerobic *Desulfovibrio desulfuricans,* also requires methylene-THF to be condensed with CO_2_ to form glycine [38] that is then converted to acetyl-CoA.

**Figure 1:**
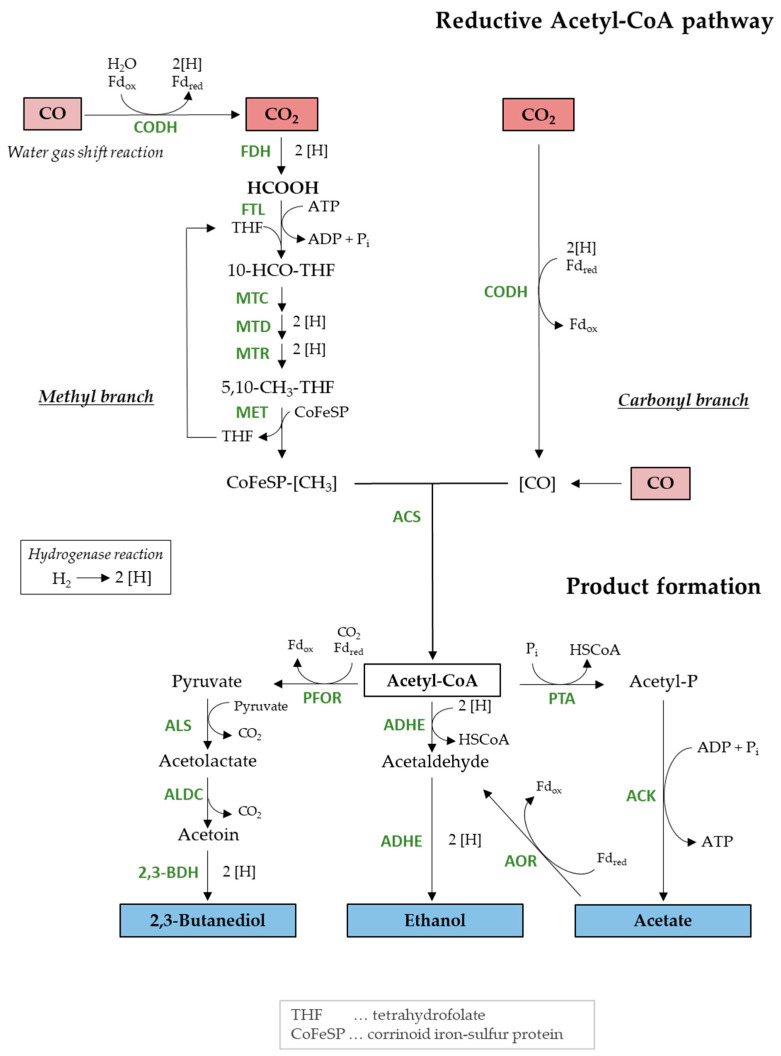
Reductive acetyl-CoA pathway (or Wood–Ljungdahl pathway) and product formation in *C. autoethanogenum*, *C. ljungdahli*, and *C. ragsdalei*. CODH: carbon monoxide dehydrogenase, FDH: formate dehydrogenase, FTL: formate-tetrahydrofolate ligase, MTC: methylene tetrahydrofolate cyclohydrolase, MTD: methylene tetrahydrofolate dehydrogenase, MTR: methylene tetrahydrofolate reductase, MET: methyltransferase, ACS: acetyl-CoA synthase, ADHE: aldehyde-alcohol dehydrogenase, AOR: aldehyde:ferredoxin oxidoreductase, ACK: acetate kinase, PTA: phosphate acetyltransferase, PFOR: pyruvate:ferredoxin oxidoreductase, ALS: acetolactate synthase, ALDC: acetolacate decarboxylase, 2,3-BDH: 2,3-butanediol dehydrogenase.

The sequential reduction of formate to methylene-THF is also present in the methyl branch of the reductive acetyl-CoA pathway, also known as Wood–Ljungdahl pathway (WLP), which is the carbon fixation pathway in acetogenic bacteria (see Figure 1). As a first step, a formate dehydrogenase catalyzes the reduction of CO_2_ to formate. Next, two sequential reactions are catalyzed by formate-tetrahydrofolate ligase (FTL) to synthesize formyl-THF under the consumption of one ATP. This reaction is followed by three further reduction reactions to methenyl-THF, methylene-THF, and finally to methyl-THF. Together with a carbonyl group delivered from CO_2_ reduction in the carbonyl branch of the WLP, the main central intermediate acetyl-CoA is formed. *Acetobacterium woodii* is an example of growth with formate using this pathway [39,40].

In gas fermentation, the growth and product formation rates of autotrophic acetogenic bacteria are often limited by the gas–liquid mass transfer limitations within the bioreactor. Therefore, acetogenic bacteria could utilize formate, which has a good water solubility, more easily as a carbon source since the low solubility of the gaseous substrates limits their availability [41]. Furthermore, the reduction degree of carbon in formate is higher than that in CO_2_. Instead of spending reduction equivalents on the CO_2_ reduction to formate in the methyl branch of the WLP, this reducing power could be saved by formate usage. The generation of the more reduced [CO] in the carbonyl branch from CO_2_ reduction also costs considerable biochemical energy equivalents due to the very low redox potential of CO_2_/CO [42,43]. Therefore, bacteria able to use CO as a sole carbon source usually prefer it over CO_2_/H_2_ for thermodynamic reasons. Nevertheless, CO_2_/H_2_ growth cultures can really profit from an additional formate or CO supply. It was shown that feeding small amounts of CO to *C. autoethanogenum* allowed a higher biomass concentration and improved bacterial fitness [44]. In another study, *A. woodi* achieved higher biomass yields when formate or CO were consumed in addition to CO_2_/H_2_ [39].

*A. woodii* has already proven its flexibility towards C1 substrate utilization, but other acetogenic bacteria are attractive in regards of more value-added natural products than acetate. In this case, the bacterium *C. ragsdalei* portrays as an opportunity to produce ethanol and 2,3-butanediol from syngas. It is known that the highest growth rates of *C. ragsdalei* are achieved at 37 °C and pH 6.3 [12]. Providing additional sulfur by sulfide feeding resulted in the highest autotrophic ethanol and 2,3-butanediol production at pH 5.5 in stirred-tank bioreactors with continuous gassing [45]. Furthermore, *C. ragsdalei* can reduce propionic and butyric acid to their corresponding alcohols 1-propanol and 1-butanol [46].

*C. ragsdalei* prefers CO as a sole carbon and electron source [12]. The parallel utilization of CO, CO_2_, H_2_, and formate has not been studied to date. Therefore, in the first part of our study, the goal was to find the initial CO partial pressure (pCO_0_) necessary to allow additional CO_2_/H_2_ consumption by *C. ragsdalei* in continuously gassed stirred-tank batch processes. The pCO was lowered stepwise in successive individual batch processes. In regards to a bio-electrochemical application where CO and formate are produced at a cathode, we focused in the second part of our study on the simultaneous formate conversion as an additional carbon and electron source. Formate was added continuously at various pCO values to study the simultaneous conversion of the C1 substrates (CO, CO_2_, and formate) and H_2_ by *C. ragsdalei* in fed-batch-operated stirred-tank bioreactors with continuous gassing. It should be answered how its consumption affects *C. ragsdalei*’s growth behavior and how the product formation alters.

## 2. Results

### 2.1. Studies on the Simultaneous Conversion of CO, CO_2_, and H_2_ by C. ragsdalei

**Figure 2:**
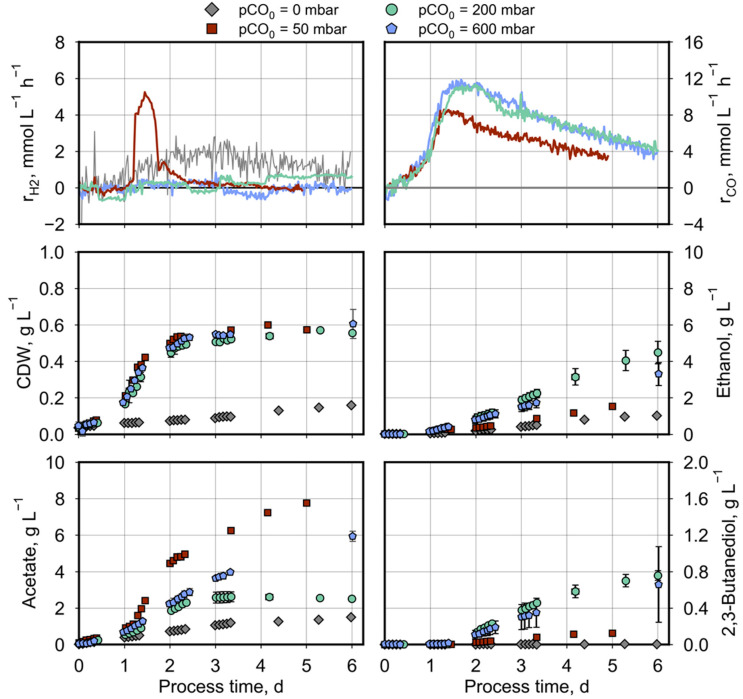
Autotrophic product formation by *C. ragsdalei* at various CO partial pressures in the incoming gas (pCO_0_). Batch processes were conducted in a fully controlled stirred-tank bioreactor (T = 32 °C, pH 5.5, n = 1200 min^−1^). The bioreactor was continuously gassed with 5 L h^−1^ of a defined gas mixture composed of 200 mbar CO_2_, 200 mbar H_2_, and 600 mbar CO/N_2_ mixtures (50 mbar CO complemented with 550 mbar N_2_, 200 mbar CO complemented with 400 mbar N_2_, and 600 mbar CO_,_ respectively). The reference process without CO was gassed with 200 mbar CO_2_ and 800 mbar H_2_.

Since pH 5.5 and 32 °C supported ethanol and 2,3-butanediol formation by *C. ragsdalei* [45], these reaction conditions were applied for all batch processes in a stirred-tank bioreactor with continuous gassing. Starting from 600 mbar CO in the synthetic syngas, pCOs of 200 mbar and 50 mbar were tested and compared to 0 mbar CO in individual autotrophic batch processes, keeping the partial pressures of H_2_ and CO_2_ (pH_2_ and pCO_2_, respectively) constant at 200 mbar by substituting CO with N_2_ gas. The product formation kinetics measured in the individual batch processes are illustrated in Figure 2.

At the initial 50–600 mbar CO, *C. ragsdalei* started to utilize CO as the only carbon source (see Figure 2). When a pCO of 30 mbar and a cell dried weight (CDW) concentration of 0.25 g L^−1^ were reached after a process time of 1.2 d, a sudden increase in the H_2_ uptake rate of up to 5 mM h^−1^ was observed (Figure 2). Meanwhile a maximal CO uptake rate of 8 mM h^−1^ was achieved and stayed constant for around 12 h, indicating CO limitation. Under excessive CO conditions (at 200 and 600 mbar), a higher CO uptake rate of 11 mM h^−1^ was measured without any hydrogen consumption (Figure 2).

The growth of *C. ragsdalei* was independent of the initial pCO_0_ within the estimation error (Figure 2), but was very much improved compared to the reference process without CO resulting in a final CDW concentration of around 0.55 g L^−1^ compared to 0.18 g L^−1^ after 6 days.

Product formation varies strongly with the initial pCO_0_ (Figure 2): Acetate formation was the highest at a pCO_0_ of 50 mbar. In particular, during H_2_ uptake, the acetate production rate increased considerably at a process time of 1.2–1.8 d. The formation of alcohols was the lowest at 50 mbar CO, with 1.5 g L^−1^ ethanol and 0.1 g L^−1^ 2,3-butanediol, after 5 days. Only in the reference process without CO, fewer alcohols were formed (1 g L^−1^ ethanol and no 2,3-butanediol).

At a pCO_0_ of 200–600 mbar, the final 2,3-butanediol concentrations (0.63–0.98 g L^−1^) did not differ significantly, but variations were observed in the ethanol and acetate production. At the pCO_0_ of 200 mbar, *C. ragsdalei* started to produce acetate, but after three days, the acetate concentration was kept constant. With final 4.2–4.8 g L^−1^ ethanol and 2.1–3.08 g L^−1^ acetate, an ethanol-to-acetate ratio of 1.6–2.0 g_EtOH_ g_acetate_^−1^ was achieved. In comparison, the acetate production continued after three process days at a pCO_0_ of 600 mbar to 5.7–6.2 g L^−1^ acetate, whereas the final ethanol concentration was reduced to 2.7–4.0 g L^−1^, resulting in an ethanol-to-acetate ratio of 0.5–0.6 g_EtOH_ g_acetate_^−1^.

**Figure 3:**
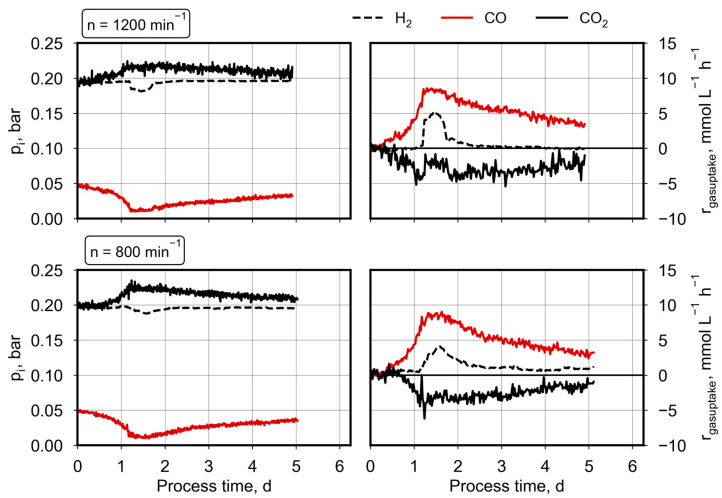
Syngas conversion by *C. ragsdalei* at an initial pCO of 50 mbar and different stirrer speeds (n). The batch processes were conducted in a fully controlled stirred-tank bioreactor (T = 32 °C, pH 5.5) with continuous gassing (5 L h^−1^, 200 mbar CO_2_, 200 mbar H_2_, 50 mbar CO, and 550 mbar N_2_).

Due to the low gas solubility of CO and H_2_, a high stirrer speed of 1200 min^−1^ was applied to allow a high mass transfer from the gas to the liquid phase by the strong power input (not determined). Another option for higher dissolved gas concentrations is the application of overpressure in general. In industry, the gas fermentation process is usually conducted in 20–30 m high bubble column reactors, where through hydrostatic pressure, high gas partial pressures are achieved at the bottom. Since the lab-scale reaction vessel in this study was not designed to withstand overpressure, a high stirrer speed was chosen. To check the possibility of enlarging the process time for simultaneous CO and H_2_/CO_2_ conversion in autotrophic batch processes with *C. ragsdalei*, an additional batch process was performed at the pCO_0_ of 50 mbar CO with a reduced stirrer speed (800 min^−1^ instead of 1200 min^−1^). Reducing the mechanical power input in the stirred-tank bioreactors resulted in reduced gas transfer rates and, thus, in reduced solved gas concentrations in the liquid phase at the same microbial gas consumption rates. The measured partial pressures of H_2_, CO_2_, and CO as well as the estimated gas uptake rates are compared in Figure 3 for the batch processes with the varying stirrer speeds.

CO consumption rates are very similar, but hydrogen uptake was observed until the end of the batch process with 800 min^−1^. The characteristic increase in the hydrogen uptake rate after 1.2 d was observed as well, but was less pronounced with a reduced mechanical power input (maximal H_2_ uptake rate of 4 mM h^−1^ compared to 5 mM h^−1^). In total, more hydrogen was consumed at a reduced stirrer speed, and consequently less CO_2_ was produced by *C. ragsdalei* in the batch process with continuous gassing (Table 1). The reduced power input did not affect the biomass or product formation (see Table 1). A direct comparison of the measured product concentrations of these two processes is presented in the Supplementary Material (see Figure S1).

From the first part of this study, two main conclusions can be drawn. For *C. ragsdalei*, the pCO_0_ had a great influence on the product distribution in autotrophic batch processes with continuous gassing. In terms of the best ethanol-to-acetate ratio, a pCO_0_ of 200 mbar CO was determined to be best, because either a higher pCO_0_ (600 mbar) or a lower pCO_0_ (50 mbar) lead to enhanced acetate formation. Further, limiting CO concentrations in the stirred tank bioreactor were necessary to allow additional CO_2_/H_2_ uptake. Looking to the future bio-electrochemical applications where CO_2_ is reduced to mainly CO and formate as by-products, the next part of our study focused on the parallel conversion of the gaseous substrates together with formate.

### 2.2. Studies on the Simultaneous Conversion of Syngas and Formate by C. ragsdalei

**Figure 4:**
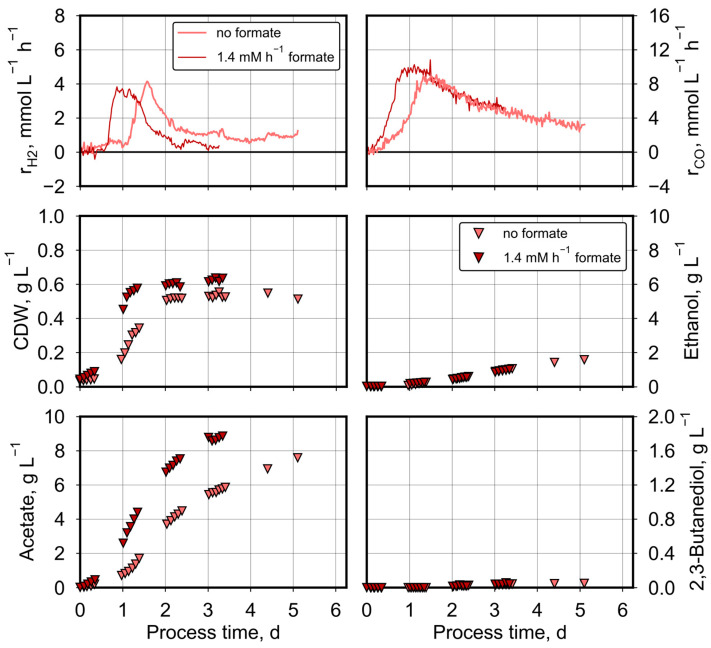
Autotrophic formate conversion by *C. ragsdalei* at the pCO_0_ of 50 mbar CO with and without formate feeding. Processes were conducted in a fully controlled stirred-tank bioreactor (T = 32 °C, pH 5.5, n = 800 min^−1^). The bioreactor was continuously gassed with 5 L h^−1^ of a defined gas mixture composed of 200 mbar CO_2_, 200 mbar H_2_, 50 mbar CO, and 550 mbar N_2_. Formate was continuously fed with 1.4 mM h^−1^.

**Figure 5:**
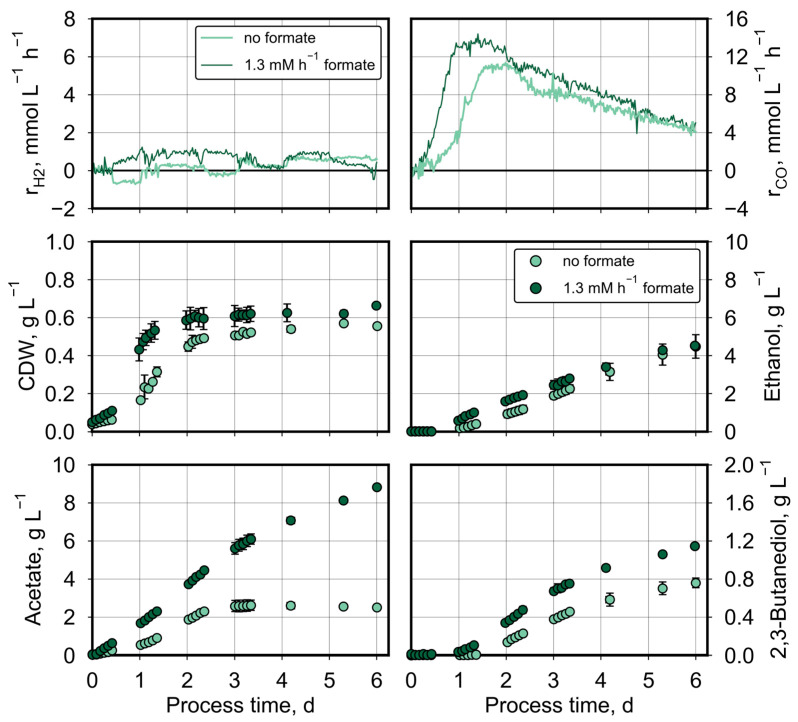
Autotrophic formate conversion by *C. ragsdalei* at the pCO_0_ of 200 mbar CO with and without formate feeding. Processes were conducted in a fully controlled stirred-tank bioreactor (T = 32 °C, pH 5.5, n = 1200 min^−1^). The bioreactor was continuously gassed with 5 L h^−1^ of a defined gas mixture composed of 200 mbar CO_2_, 200 mbar H_2_, 200 mbar CO, and 400 mbar N_2_. Formate was continuously fed with 1.3 mM h^−1^. All processes were reproduced. The mean with min-max values are shown for the concentration data, whereas the gas uptake rates r_i_ are presented only for one of the duplicated processes.

**Figure 6:**
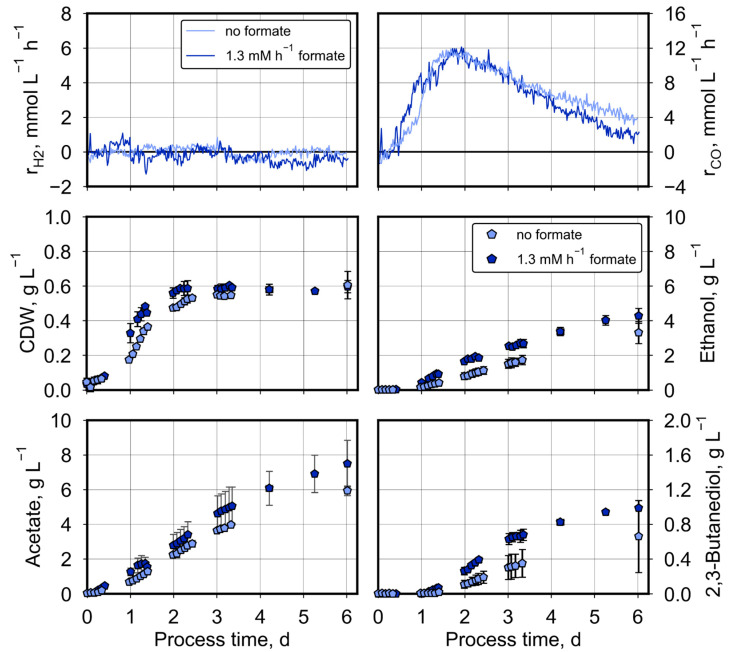
Autotrophic formate conversion by *C. ragsdalei* at the pCO_0_ of 600 mbar CO with and without formate feeding. Processes were conducted in a fully controlled stirred-tank bioreactor (T = 32 °C, pH 5.5, n = 1200 min^−1^). The bioreactor was continuously gassed with 5 L h^−1^ of a defined gas mixture composed of 200 mbar CO_2_, 200 mbar H_2_, and 600 mbar CO. Formate was continuously fed with 1.3 mM h^−1^. All processes were reproduced. The mean with min-max values are shown for the concentration data, whereas the gas uptake rates r_i_ are presented only for one of the duplicated processes.

Continuous formate feeding of 1.2–1.4 mM h^−1^ was applied at varying pCO_0_ values in the syngas (50–600 mbar CO) in the stirred-tank reactors. The process performances were compared to the corresponding batch processes without formate supply (Figure 4, Figure 5 and Figure 6). The displayed concentration data represent the mean values of two individual processes with continuous gassing at the same process conditions, with the exception of the processes at pCO_0_ of 50 mbar CO. The gas uptake data are depicted from one of the duplicated processes with and without formate feeding. Due to the constant formate feeding rate, formate accumulated at the beginning at low cell densities. When a CDW concentration of about 0.05 g L^−1^ was reached, all the formate in the liquid medium was taken up by *C. ragsdalei*. Until the end of the fed-batch processes, no formate was detected by HPLC anymore.

At a pCO_0_ of 50 mbar CO and a reduced stirrer speed of 800 min^−1^, parallel formate, CO, CO_2_, and H_2_ conversion was observed (Figure 4). Hydrogen consumption was initiated earlier at a process time of 0.6 days with additional formate feeding. CO consumption as well as the growth of *C. ragsdalei* and acetate formation were clearly improved with formate feeding, whereas no differences in process performances were measured with respect to microbial ethanol and 2,3-butanediol production.

Compared to the autotrophic batch process, additional formate feeding raised both the total amount of consumed CO from 401 mmol to 508 mmol and produced CO_2_ from 179 mmol to 285 mmol. Carbon balances and electron balances were closed within the estimation error as before, with a carbon recovery of 104–105% and an electron recovery of 109–113% (Table 2).

Applying a higher pCO_0_ of 200 mbar CO and continuous formate feeding showed the same effects as before (Figure 5): increased CO consumption, increased growth rate of *C. ragsdalei*, enhancedacetate formation and no differences in ethanol formation compared to the corresponding autotrophic batch process without formate feeding. In contrast to the autotrophic fed-batch process with 50 mbar CO shown before, the microbial 2,3-butanediol production was clearly improved with formate feeding (1.15 ± 0.02 g L^−1^ compared to 0.76 ± 0.13 g L^−1^).

Compared to the autotrophic batch process at a pCO_0_ of 200 mbar CO, additional formate feeding raised again both the total amount of consumed CO from 710 ± 21 mmol to 990 ± 1 mmol and produced CO_2_ from 455 ± 18 mmol to 636 ± 3 mmol (Table 3). Carbon balances and electron balances were closed within the estimation error as before, with a carbon recovery of 95–97% and an electron recovery of 91–94%. Integrating hydrogen gas consumption over the whole process time resulted in an estimated total uptake of small amounts of 94–136 mmol H_2_ within 6 days, whereas no net H_2_ uptake was observed in the batch process without formate feeding.

In contrast to the data shown before, no significant differences in process performances were observed at a pCO_0_ of 600 mbar CO with and without formate feeding (Figure 6). Though the mean of the concentration data shows slightly increased product concentrations, the min-max values of the duplicated processes were two high to indicate significance. Carbon balances and electron balances were closed within the estimation error as before, with a carbon recovery of 97–103% and an electron recovery of 106–110% (Table 3). The share of carbon fed as formate and consumed by *C. ragsdalei* in relation to total carbon conversion was 12–16% at 600 mbar CO. As this was not significantly different from the respective carbon share of 11–13% at a pCO_0_ of 200 mbar, we assumed that the improved availability of CO for *C. ragsdalei* cells at 600 mbar CO may be the reason for the non-significant effects of formate feeding with 1.3 mM h^−1^.

**Figure 7:**
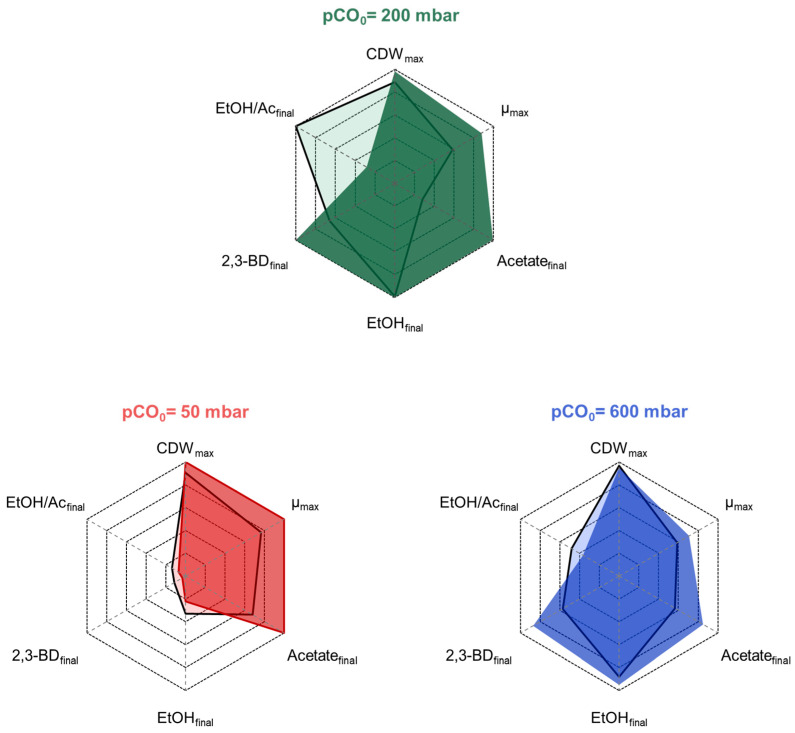
Relative effect of formate feeding on CDW_max_, μ_max_, the final ethanol-to-acetate ratio (EtOH/Ac_final_), and the relative final product concentrations (acetate, ethanol, and 2,3-butanediol) at the initial 50, 200, and 600 mbar CO. Processes were conducted in a fully controlled stirred-tank bioreactor (T = 32 °C, pH 5.5, n = 1200 min^−1^). The formate feed rate was 1.4 mM h^−1^ at a pCO_0_ of 50 mbar and 1.3 mM h^−1^ at 200 and 600 mbar CO. The black lines present the data without formate feeding at the respective pCOs.

Summarizing the results from these investigations, simultaneous formate consumption is possible in stirred-tank bioreactors with continuous syngas gassing at the rates studied independently of the pCO_0_ tested. At the lower pCO_0_ of 50–200 mbar CO, formate addition boosted growth and product formation with *C. ragsdalei*, whereas no significant effects were observed with 600 mbar CO in the syngas (Figure 7).

## 3. Discussion

### 3.1. A Limiting pCO Induced Simultaneous Conversion of CO, CO_2_, and H_2_ by C. ragsdalei

With the aim of complete carbon conversion, the parallel consumption of all components of a synthesis gas mixture is crucial. Therefore, autotrophic batch processes were conducted with *C. ragsdalei* to identify the process conditions for the simultaneous conversion of CO, CO_2_, and H_2_ by varying the pCO_0_ in the inlet gas of a stirred-tank bioreactor with continuous gassing.

When CO was limited at around 30 mbar in the reactor, additional CO_2_ and H_2_ uptake satisfied the demand for gaseous carbon and electrons to achieve the maximal biomass concentration in batch processes. The CO limitation was reflected by the reduced CO uptake rate in comparison to the rates achieved at 200 and 600 mbar CO, and the rate was constant for half a day during CO_2_/H_2_ uptake. *C. ragsdalei* is known to prefer CO as a carbon and electron source over CO_2_/H_2_ [12]. In addition, CO using acetogenic microorganisms usually favor CO over CO_2_/H_2_ as a carbon and electron source for thermodynamic reasons [47]. Missing Fd_red_ as a reducing equivalent that is mainly generated from the CO to CO_2_ oxidation (see Figure 1) causes ATP limitation since the ATPase activity is dependent on the Fd_red_ pool [48]. Besides, a strong acetate production decouples the proton motive force, resulting in additional ATP costs [49]. Coincidently with H_2_ uptake, the acetate production was raised to produce more ATP, but missing reducing equivalents restricted the further conversion to ethanol and the production of 2,3-butanediol.

In the study of Heffernan et al. (2020)*,* CO was supplemented in a CO_2_/H_2_ grown *C. autoethanogenum* culture with a share of 2% that was lower than that in our study (5%), and it was shown that the CO supplementation boosted the biomass production, enabled a higher ethanol-to-acetate conversion, and demonstrated parallel CO and CO_2_ conversion [44]. In *C. autoethanogenum*, the high ATP demand was satisfied by an increased CO oxidation to elevate the Fd_red_ and consequently the ATP pool [49]. For *C. ragsdalei* under limiting CO conditions, this mechanism could not be employed, resulting in an aggravated ethanol-to-acetate ratio. However, the simultaneous conversion of CO, CO_2_, and H_2_ by *C. ragsdalei* was proven in this study.

### 3.2. The pCO Affected the Product Distribution

The batch studies performed with *C. ragsdalei* in a fully controlled stirred-tank bioreactor with continuous gassing showed that the acetate, ethanol, and 2,3-butanediol production rates of this bacterium were highly affected by the pCO value (Figure 7).

*C. ragsdalei* grew very slowly without CO, but this might be improved by a pH increase, since its optimum value for growth is pH 6.3 [12], and throughout this study, a pH of 5.5 was applied. Product formation from CO_2_/H_2_ did not enable 2,3-butanediol production, but the ethanol-to-acetate ratio (0.7 g_EtOH_ g_acetate_^−1^) was higher than in the process conducted at 50 mbar CO (0.2 g_EtOH_ g_acetate_^−1^), where *C. ragsdalei* grew solely with CO first and started to take up CO_2_/H_2_ later. Comparing the growth without CO to *C. ljungdahlii*, like in the study from Hermann et al. (2020), mainly biomass and acetate were produced at pH 5.9 as well, and ethanol production even started decoupled from growth only at the end of the batch process [50].

When *C. ragsdalei* grew solely with CO, a pCO of 200 mbar resulted in the highest ethanol-to-acetate ratio, as a direct conversion of acetate into ethanol was observed. This is possible via acetaldehyde by the aldehyde ferredoxin oxidoreductase (AOR, see Figure 1). That ethanol is rather synthesized via this route and not directly from acetyl-CoA was also reported for *C. autoethanogenum* [51] and for *C. ljungdahlii* [52] as overflow metabolism, in the latter case explained by an excess of acetyl-CoA and reduction equivalents, such as ferredoxin, which cannot be used for biomass formation but are forwarded to alcohol production. For *C. ragsdalei*, the reduction from pH 6.0 to pH 5.5 confirmed the beneficial influence on ethanol and 2,3-butanediol production [45], but in our study, an optimal pCO was found to maximize the alcohol production. It should be mentioned that, in the study from Oliveira et al. [45], the acetate-to-ethanol conversion was observed at a pCO of 600 mbar with a reduced stirrer speed (800 min^−1^ instead of 1200 min^−1^ in our study), affecting therefore the real (not measured) dissolved CO concentration in the liquid phase.

Remarkably, at 600 mbar CO, little hydrogen production was observed throughout the process, indicating a disposal of excess reducing equivalents instead of redirecting the reducing power towards ethanol and 2,3-butanediol in terms of redox homeostasis.

### 3.3. Parallel Utilization of Formate, CO, CO_2_, and H_2_

Formate as a stable C1 product from electrocatalytical CO_2_ reduction gained considerable attention. Acetogenic bacteria natively dispose one of the most straightforward carbon fixation pathways, the reductive Acetyl-CoA pathway, which is energetically very efficient at the same time and allows the direct utilization of formate as a further C1 carbon and electron source. Therefore, electrocatalytical formate formation was simulated physically by a continuous formate feed. A parallel CO and CO_2_/H_2_ uptake had been already demonstrated at an initial pCO_0_ of 50 mbar, but a parallel formate consumption at various pCO (50, 200, and 600 mbar) was the next research question to be investigated at moderate formate supply rates (1.3–1.4 mM h^−1^).

The simultaneous conversion of CO, CO_2_, H_2_, and formate was observed only at a pCO_0_ of 50 mbar. The formate supply did not affect the H_2_ uptake, but an increased total CO uptake was measured. Carbon recoveries proved that formate was utilized as an additional carbon and electron source and did not substitute part of the CO or CO_2_/H_2_. An increased flux through the methyl branch in the WLP might explain the enhanced CO uptake to deliver the needed carbonyl group to produce 1 acetyl-CoA (see Figure 1). The enhanced flux enabled a higher growth rate by 20–50%, independent on the initial pCO_0_. A deficient alcohol production under CO limitation was shown to be one characteristic of *C. ragsdalei*, and additional formate feeding increased the synthesis of the less attractive fermentation product acetate and worsened the 2,3-butanediol production. However, a stronger acetate production was observed independent of the incoming pCO_0_. The enhanced ATP demand of the ATP-consuming formate fixation step in the methyl branch was compensated by acetate production, impairing the ethanol-to-acetate ratio (see Figure 1).

Remarkably, at a pCO_0_ of 200 mbar, 2,3-butanediol production was boosted by formate feeding (Figure 5). Considering the carbon in formate, the carbon enters the methyl branch in a more reduced state compared to CO_2_. Accordingly, *C. ragsdalei* can save reduction equivalents (2H^+^ + 2e^−^) through preventing the required reduction of CO_2_ to formate by the formate dehydrogenase, in theory. If ethanol is mainly synthesized through the acetate route, these energy savings were useful in the production of 2,3-butanediol, which needs 2 NADH as reducing equivalents (see Figure 1). But the improved 2,3-butanediol synthesis was dependent on a high CO availability since at limiting CO concentrations, *C. ragsdalei* mainly synthesized acetate.

Among different microorganisms, only few can utilize formate. For example, *Acetobacterium woodii* is known for its good CO_2_/H_2_ utilization as the main carbon and electron sources, but can be very flexible towards autotrophic and heterotrophic substrates [39]. An *A. woodii* culture, grown with CO_2_/H_2_, was supplemented with formate. Although the reduced gas uptake rates indicated that formate substituted a part of the total consumed carbon, higher biomass yields could be achieved [39]. Interestingly, independent of the used carbon and electron sources, the final acetate yields based on the total carbon consumed did not differ significantly between the *A. woodi* cultures grown with CO_2_/H_2_ or CO_2_/H_2_/formate or CO_2_/H_2_/formate/CO, showing that the additional energy delivered by CO was rather used for biomass production. In the end, the co-utilization of CO, formate, and CO_2_/H_2_ by *A. woodii* resulted in higher biomass and acetate concentrations, similar to *C. ragsdalei*, although *A. woodi* is not an alcohol producer.

## 4. Materials and Methods

### 4.1. Microorganism and Media

The acetogen *Clostridium ragsdalei,* obtained from the German Collection of Microorganism and Cell Cultures GmbH (DSM No. 15248, DSMZ GmbH, Braunschweig, Germany) as a freeze-dried sample, was grown in a fermentation medium and then stored at −80 °C with 10% glycerol (*v*/*v*). The fermentation medium for pre-cultivation and batch processes in the stirred-tank bioreactor was composed according to Doll et al. [53], and the ingredients are listed in the Supplementary Material (Table S1). For pre-cultivation, 15 g L^−1^ 2-(N-morpholino)ethanesulfonic acid (MES) was added as a buffer. A total of 2 L of the freshly prepared fermentation medium for pre-cultivation was boiled for 20 min and strongly purged with N_2_ for at least 20 min for anaerobization. 100 mL of the medium was aliquoted to 0.5 L anaerobic bottles in an anaerobic glove box and then autoclaved. Chemical stocks (fructose, cysteine-hydrochloride, and PBS buffer) were made with anaerobic water and autoclaved for sterilization.

### 4.2. Pre-Cultivation and Inoculum Preparation

The pre-cultures were incubated at 37 °C and 100 rpm (WiseCube WIS-20, Witeg Labortechnik GmbH, Wertheim, Germany). The first heterotrophic pre-culture (7 g L^−1^ fructose) grew over 70 h and was used to inoculate a second autotrophic pre-culture with 10 mL (gas mixture: 1.2 bar CO, 0.4 bar CO_2_, and 0.4 bar H_2_). The second pre-culture contained 0.4 g L^−1^ of cysteine hydrochloride as a reducing agent and served as the inoculum for the batch process. The stirred-tank bioreactor was always inoculated in a way to achieve a starting CDW concentration of around 0.03 g L^−1^. For the main inoculum, the second grown culture was harvested by centrifugation at 3600 rcf (Rotixa 50 RS, Hettich GmbH & Co., KG, Tuttlingen, Germany), and the cell pellet was resuspended in 10 mL of anaerobic PBS buffer.

### 4.3. Continuously Gassed Stirred-Tank Bioreactor

Batch processes were performed in a stirred-tank bioreactor. The cylindrical reaction vessel was designed previously for bio-electrochemical applications [27]. The processes were conducted with a 1 L working volume under fully controlled conditions. The temperature and pH were adjusted to 32 °C and pH 5.5, respectively, according to Oliveira et al. [45], using a control station (Labfors 2, Infors AG, Bottmingen, Switzerland). The pH was measured by a sterizable sensor (405-DPAS-SC-K8s/120, Mettler Toledo, Giesen, Germany). The bioreactor was continuously gassed with a defined gas mixture at ambient pressure. Each gas volume flow was controlled by an independent mass flow controller (F-201CV-500 RGD-33-V, Bronhorst High Tech B.V., Ruurlo, the Netherlands) and mixed before reaching the inlet of the bioreactor. The gas composition always comprised 20% CO_2_ and 20% H_2_. The CO share was varied in the range of 5–60%, whereas at lower partial pressures than 600 mbar CO, the mixture was complemented with N_2_ to achieve a constant gas volume flow of 5 NL h^−1^. The stirred-tank bioreactor was equipped with baffles and two Rushton turbines. A stirrer speed of n = 1200 min^−1^ was applied. Both the bioreactor and fermentation medium without vitamins were autoclaved separately. The medium was transferred to the bioreactor under sterile conditions. Sterile filtrated vitamins (0.2 µm pore size) were added to the medium that was anaerobized with 5 NL h^−1^ N_2_ for around 2 h. Afterwards, the bioreactor was calibrated with the defined gas mixture over night. Before the inoculation with *C. ragsdalei*, 0.4 g of L^−1^ cysteine hydrochloride was added aseptically as a reducing agent to generate a redox potential below −100 mV.

To simulate electrocatalytical formate production physically, a continuous formate feed was applied using a 2.5 M sodium formate solution (pH 5.5) with a feed rate of 0.5 mL h^−1^. The feed was started concurrently with the inoculation. The weight of the stock solution was also monitored and allowed for the determination of the real applied formate feed rate by linear regression at the end. Due to sampling and the very slow feeding rate, dilution was neglectable.

### 4.4. Analytical Methods

During the batch processes, samples were collected regularly from the bioreactor and analyzed for biomass and product concentrations. The bacterial growth was monitored through optical density measurements at 600 nm (OD_600_). Afterwards, a previously determined correlation between the OD_600_ and CDW concentration was used to calculate the CDW for the corresponding measurements. The samples were filtered (0.2 μm size exclusion), and the supernatant was analyzed for organic acids and alcohols by HPLC (LC2030C-Plus, Shimadzu, Kyoto, Japan) using a carbohydrate analysis column (Aminex HPX-87H, Biorad, Munich, Germany) at 60 °C. The analytes were eluted isocratically using 5 mM of H_2_SO_4_ as the mobile phase at a flow rate of 0.6 mL min^−1^. Detection was achieved by a refractive index detector (RID-20A, Shimadzu, Kyoto, Japan), and an external standard with defined analyte concentrations allowed for their quantification. The lowest concentrations in the calibration curves were 0.1 g L^−1^ of ethanol, 0.04 g L^−1^ of formate, 0.2 g L^−1^ of acetate, and 0.04 g L^−1^ of 2,3-butanediol. Through sigmoidal model fittings by non-linear regression using the Gompertz curve [54], time-dependent product formation rates were estimated. To calculate the maximal specific growth rate, the CDW concentration curve was fitted to the logistic growth function by Richards F.J. [55]. The derivative of the model was related to the initial CDW concentration to calculate the growth rate at each time point.

The off-gas of the bioreactor was continuously measured by a mass flow meter (F-111B-500-RGD-33-E, Bronkhorst High Tech B.V., Ruurlo, The Netherlands) and analyzed online by gaschromatography every 10 min. The micro-gaschromatograph (590 Micro GC, Agilent Technologies, Waldbronn, Germany) was equipped with a COX column for the retention of H_2_, CO, and CO_2_, which were detected by thermal conductivity. External calibration with the defined gas mixtures allowed for the determination of the respective partial pressures or gas shares in the exhaust gas. N_2_ could not be measured and was calculated by subtracting the determined partial pressures from the absolute pressure. The off-gas mass flow was corrected by the mixing gas conversion factor that included the gas shares and the individual linear calibration parameters of each analyzed gas. Then, the corrected volume flow was multiplied by the gas shares to estimate the individual gas volume flows for each GC measurement. Subtracting the measured volume flow from the initial volume flow allowed for the calculation of the gas uptake rates. Further integration over time, using a 10 min increment, delivered the total gas consumption in mmol.

### 4.5. Carbon and Electron Recoveries

The measured final product concentrations in [g L^−1^] were converted to [mmol C L^−1^]. The total amount of products (CDW, ethanol, acetate, 2,3-butanediol, and CO_2_) was then related to the total consumed carbon (CO, yeast extract, and formate) to calculate the carbon recoveries. For determining the electron recovery, the respective reduction degrees of each molecule were used and H_2_ was also considered.

## 5. Conclusions

In electrochemical systems, the main goal is to achieve high faradaic and energy efficiencies. Biotransformation processes using microorganisms can allow the direct conversion of electrochemical products into valuable molecules. In case of the electrocatalytical CO_2_ reduction reaction (CO_2_RR) in a bioreactor, formate can be synthesized as a by-product beside CO, and H_2_ gas usually evolves from the competing proton reduction reaction in the aqueous system. Depending on the applied cell potential, the electrochemical product distribution can vary. The evidence of simultaneous CO, CO_2_, H_2_, and formate utilization by *C. ragsdalei* was provided. In regards of a bio-electrocatalytical system in which all these C1 substrates and H_2_ are accessible in various stoichiometries, a parallel conversion will be necessary for high carbon and energy efficiency. Using electric energy, generated by solar panels or windmills, and CO_2_ derived from fossil fuels, a sustainable process can be developed in the future. In this case, *C. ragsdalei* is a candidate for bioelectrocatalytical acetate and ethanol production from CO, formate, CO_2_, and H_2_. In industrial-scale gas fermentation without electrochemical application, the process with *C. ragsdalei* can be conducted in high bubble column reactors. At the bottom of the column, a high CO partial pressure enables a high CO availability, whereas at the top, a low CO concentration will allow parallel CO, CO_2_, and H_2_ conversion. Co-cultivation with chain-elongating anaerobic microorganisms, like *C. kluyveri*, in a bio-electrochemical system may allow the assimilation of acetate and ethanol to produce longer-chained organic acids and alcohols and should be investigated in future studies.

## Figures and Tables

**Table 1:** Integral carbon and electron balances of CO limited autotrophic batch processes at initial 50 mbar CO with *C. ragsdalei* at varying stirrer speeds (batch-operated stirred-tank bioreactors with continuous gassing: 32 °C, pH 5.5, and 5 NL h^−1^ syngas with 200 mbar CO_2_, 200 mbar H_2_, 50 mbar CO, and 550 mbar N_2_).

	Stirrer Speed n, min^−1^	1200	800
Substrates, mmol C	CO	507	505
	Yeast extract	9	9
Products, mmol C	Biomass	18	18
	Ethanol	51	62
	2,3-Butanediol	5	2
	Acetate	233	231
	CO_2_	276	230
**Carbon recovery, %**		114	106
**Electron recovery, %**		117	110
H_2_ as substrate, mmol		74	128

**Table 2:** Integral carbon and electron balances of CO-limited autotrophic batch processes with and without formate feeding at an initial 50 mbar CO with *C. ragsdalei* (batch-operated stirred-tank bioreactors with continuous gassing: 32 °C, pH 5.5, 5 NL h^−1^ syngas with 200 mbar CO_2_, 200 mbar H_2_, 50 mbar CO, and 550 mbar N_2_).

Substrates, mmol C	Formate	0	110
	CO	401	508
	Medium	9	9
Products, mmol C	Biomass	18	20
	Ethanol	43	48
	2,3-Butanediol	2	2
	Acetate	190	310
	CO_2_	179	285
**C in formate/total C consumed, %**		0	18
**Carbonrecovery, %**		105	104
**Electron recovery, %**		109	113
H_2_ as substrate, mmol		108	94

**Table 3:** Integral carbon and electron balances of autotrophic batch processes with and without formate feeding at the initial 200 and 600 mbar CO with *C. ragsdalei.* Batch-operated stirred-tank bioreactors with continuous gassing: 32 °C, pH 5.5, and 5 NL h^−1^ syngas with 200 mbar CO_2_, 200 mbar H_2_, 200 (or 600) mbar CO, and 400 (or 0) mbar N_2_. The carbon and electron balances were calculated for each individual process until t = 4.4 d and allowed the presentation of minimal and maximal values. If a data point was not available, the value was calculated using a sigmoidal model (see the Methods Section).

	pCO, mbar	200		600	
Substrates, mmol C	Formate	0	124–143	0	140–141
	CO	689–731	989–991	733–919	780–1039
	Medium	10	10	10	10
Products, mmol C	Biomass	17–18	19–21	17–18	18–19
	Ethanol	115–158	140–164	91–127	136–155
	2,3-Butanediol	22–35	40–42	10–36	36–37
	Acetate	82–91	231–245	162–171	199–230
	CO_2_	437–473	633–639	501–515	510–776
**C in Formate/total C consumed, %**		0	11–13	0	12–16
**C recovery, %**		98–103	95–97	94–105	97–103
**Electron recovery, %**		92–106	91–94	92–99	106–110
H_2_ as substrate, mmol		3–6	94–136	0–20	0–112

## Data Availability

The original contributions presented in the study are included in the article/Supplementary Material; further inquiries can be directed to the corresponding authors.

## Supplementary Materials

The following supporting information can be downloaded at: https://www.mdpi.com/article/10.3390/molecules29112661/s1. Figure S1: Product formation by *C. ragsdalei* at pCO_0_ = 50 mbar at different stirrer speeds. The batch processes were conducted in a fully controlled stirred-tank bioreactor (T = 32°C, pH 5.5) with continuous gassing (5 L h^−1^, 200 mbar CO_2_, 200 mbar H_2_, 50 mbar CO, and 550 mbar N_2_). Table S1: Medium composition according to Doll et al. (2018) [53]. The mineral, vitamin, and trace elements stock solutions were prepared separately.
