# Supplementary material for: Simultaneous Formate and Syngas Conversion Boosts Growth and Product Formation by Clostridium ragsdalei
Source: Molecules. 2024 Jun 4;29(11):2661. doi: 10.3390/molecules29112661 (PMC11174074; doi:10.3390/molecules29112661)
Supplement: Supplementary file 1 [file molecules-29-02661-s001.zip › molecules-2997354-supplementary.pdf]

Simultaneous formate and syngas coversion boosts growth and product formation by *Clostridium ragsdalei*

Irina Schwarz, Angelina Angelina , Philip Hambrock and Dirk Weuster-Botz

Supplemental information

1. Product formation by *C. ragsdalei* at 50 mbar CO using different stirrer speeds

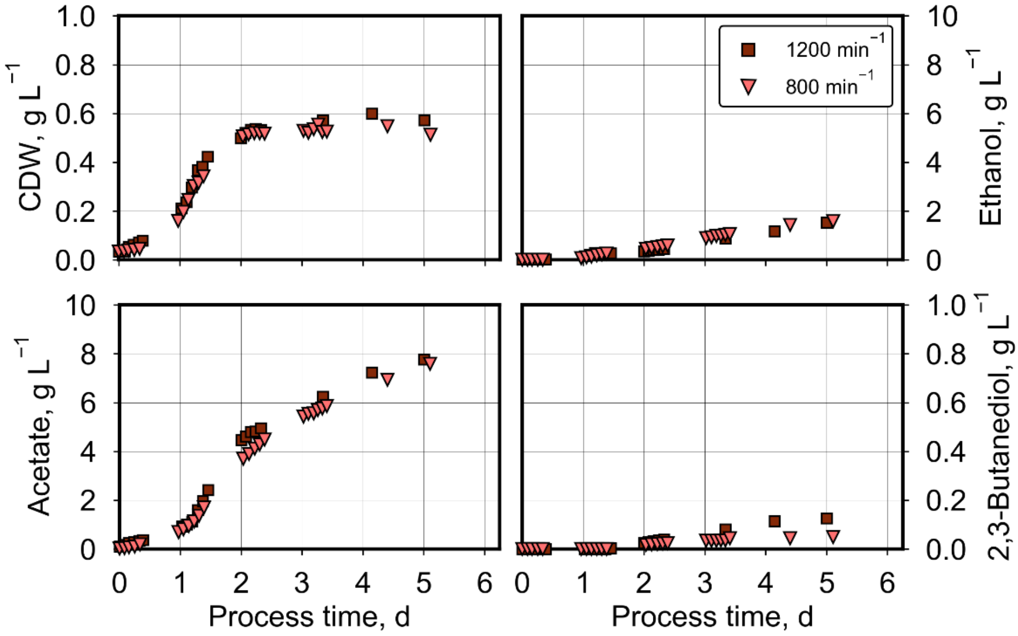

Figure S1: Product formation by *C. ragsdalei* at pCO_0 = 50 mbar at different stirrer speeds. The batch processes were conducted in a fully controlled stirred-tank bioreactor (T = 32°C, pH 5.5) with continuous gassing (5 L h^{-1}, 200 mbar CO_2, 200 mbar H_2, 50 mbar CO, and 550 mbar N_2).

2. Medium composition

Table S1: Medium composition according to Doll et al. (2018). The mineral, vitamin and trace elements stock solutions were prepared separately.

**Minerals**	g L^{-1}
NaCl	2.4
NH_4Cl	3.0
KCl	0.3
KH_2PO_4	0.3
**Vitamins**	mg L^{-1}
Pyridoxine hydrochloride	0.12
Thiamine hydrochloride	0.06
Riboflavin	0.05
Calcium pantothenate	0.05
Lipoic acid	0.05
p-Aminobenzoic acid	0.05
Nicotinic acid	0.05
Cobalamin	0.05
D-Biotin	0.02
Folic acid	0.02
Sodium 2-mercaptoethanesulfonate	0.23
**Trace elements**	mg L^{-1}
Nitrilotriacetic acid	20.0
$MnSO_4 \cdot H_2O$	11.2
$(NH_4)_2Fe(SO_4)_2 \cdot 6\ H_2O$	11.0
$Cl_2Co \cdot 6\ H_2O$	3.70
$ZnSO_4 \cdot 7\ H_2O$	3.60
$CuCl_2 \cdot 2\ H_2O$	0.25
$NiCl_2 \cdot 6\ H_2O$	0.37
$Na_2MoO_4 \cdot 2\ H_2O$	0.23
Na_2SeO_4	0.20
$Na_2WO_4 \cdot 2\ H_2O$	0.22
Yeast extract	1.00
$CaCl_2$ ▪ 2 H_2O	0.15
$MgSO_4$ ▪ 7 H_2O	1.23

References

Doll, K.; Rückel, A.; Kämpf, P.; Wende, M.; Weuster-Botz, D. Two stirred-tank bioreactors in series enable continuous production of alcohols from carbon monoxide with Clostridium carboxidivorans. *Bioprocess Biosyst. Eng.* **2018**, *41*, 1403–1416, doi:10.1007/s00449-018-1969-1.
